# Cynanche Trachealis and Crural Phlebitis

**Published:** 1871-08

**Authors:** L. B. Cotes

**Affiliations:** Batavia


VOL. XI. AUGUST, 1871. No. 1.

### Original Communications.

ART. I.—*Cynanche Trachealis and Crural Phlebitis.* By L. B. COTES, M. D., Batavia.

It is a singular fact, and one that cannot be explained, that specific epidemics have specific influences and specific localities in the human system, and what is most remarkable, that it may be confined to so small a space; the mucous membrane lining the air passages, is attacked at one time at one point and then at another, every variety of cynanche at different times.

The term croup is applied to that species of cynanche which lies between the bronchi and the larynx, sometimes over-reaching these points. The acute form of this disease as occurring in children is the most grave, but notwithstanding its acknowledged gravity through these many centuries, no one well defined mode of treatment has stood the test of experience. We are constantly presented with something new or pretending to be new, and no fixed plan that I am aware of is adopted by the profession.

The pathology of this malady is sufficiently understood, if not sufficiently studied, and kept in view in the treatment.

We are presented with a child from eight months to six years old more or less, as the case may be, fleshy or not, with hot, dry or moist surface, face red or pale, as the case may be, hurried respiration, pulse 130 to 160, great thirst, stridulous or barking cough, in short, with unmistakable indications of dynamic disease.

With this view and under these circumstances, I treated the disease with the lancet, triple salt of antimony, hot vapor and fomentations, over twenty years, adding tonics in the intermittent and remittent forms; this treatment was followed perhaps with ordinary success; at the same time, I confess, that I was in some quandary, as others seemed to be then and now, because I could not cure more of my patients; for the last sixteen years I have been more successful.

In 1854, my eyes fell upon the first article that I had seen from Dr. Norwood in relation to the medicinal properties and uses of Veratrum Viride. I was forcibly struck with it, and having a case of crural phlebitis in its inception, making rapid strides, I wrote to him, and received promptly by mail ℥ij of his tincture, At this time I found my patient, three miles away, with limb painful and swollen from body to toes, high fever, with pulse 140, knotty tense painful cord following the direction of the vessels, etc. My patient had been bled, catharticised, and was then taking antimony, digitalis and anodynes, with only temporary benefit.

I directed tinc. veratrum viride gtt. vi. to be given every three hours until I shouldvisit her at eight o'clock next morning, if vomiting or other marked effect should not be produced. Unfortunately I was called away before that hour four miles in the opposite direction, and was not able to see her until 3½ p. m., when I found her without pain, calm, cool, pulse at fifty, and very pale, felt no other inconvenience than a little prostration, she could even move her limb slightly, which she had not been able to do for several days; gave her an anodyne with directions to resume the medicine, giving three every three hours when the pulse began to come up.

Without further particulars, I will simply state, that from this time, under the continued influence of the hellebore for two weeks, I had the satisfaction of seeing the fever and inflammation vanish, and Mrs. T—, my *prima para*, go on to get well, and no permanent injury of the limb followed.

It was not long after this, (a thing that I had much coveted) before I was called to a case of membranous croup, well formed, with symptoms above alluded to, a child of 19 months, with pulse 160. I commenced with the veratrum and repeated the doses every hour until emesis was produced, this reduced the pulse to about 80, and the fever very much reduced. As soon as these began to return, the use of the remedy was resumed and continued; in a few hours my patient began to expectorate tenacious shreddy mucous, clearly indicating what was going on; the child rapidly recovered with free expectoration.

I need hardly say that my plan in the future use of this remedy was more fully formed, and I commenced an extensive use of it, and resorted to it wherever I found increased heat and pulse; and although it is a remedy of acknowledged potency, it is not, like others of this class, frought with danger in the hands of the scientific physician. I have used it in croup where the membrane was being formed, (being called late, or in council,) holding the pulse and fever, and carrying with it the use of mercury, until the membrane was thrown off, and in one instance, its expulsion came near suffocation.

In acute rheumatism, pleuro-pneumonia, &c., it is of undoubted efficacy.

In sub-acute forms of disease, it can not be used with advantage so extensively, as in typhoid fever, scarlatina, diphtheria, &c., but in very many of these it may be used in the first stage with benefit where there is a putrescent tendency from inception, it is evidently contra-indicated. At the same time, in many of those cases, where serious complications are threatened, and where aconite or antimony in $\frac{1}{16}$ to $\frac{1}{20}$ grs., to the adult every four or six hours, and less to the child, is admissable, veratrum may be employed to advantage, reducing the pulse to between 70 and 80, and holding it there until a decided amelioration in the more urgent symptoms is produced; often the skin becomes relaxed, diaphoresis supervenes, quite resembling a crisis, and I have no doubt often lessens the duration of disease.

It should be borne in mind, that to administer a few doses of veratrum and relinquish it, we shall come short of realizing our purpose; it must be continued until our patient is cured or placed on a safe footing.

If occasionally alarming prostration seems to threaten, stimulants will support and sustain; sometimes it produces an irritant effect upon the nervous systen and mucous membranes ; an anodyne will soften this.

Much has been written upon the medicinal use and properties of this article, but as far as my limited observation enables me to judge, it does not occupy the high place it merits in the treatment of the diseases indicated in this heading.

Comparatively little or nothing has been written of its use in crural phlebitis. My experience of sixteen years duration with it convinces me fully of the power of veratrum, to cut short this poisonous malady, when early and persistently used with appropriate adjuvants. I have had some severe cases, and some of which I apprehend would have been followed with permanent injury to the limb without it.

So sanguine am I of its beneficial influence in controlling the progress of these two diseases, that I do not hesitate to invite the personal experience of medical brethren to a thorough trial, not doubting but their reports will be quite satisfactory, showing a less per centage of fatal and badly cured cases.

In pneumonia, pleuro-pneumonia, &c., it is equally potent, and so much has been said in relation to its successful administration here, that I simply take this opportunity of recording my testimony with others in relation to this fact, hoping to see much more said upon its successful use, especially in discussions where *croup* is introduced before medical bodies.

